# EGFR Exon-Level Biomarkers of the Response to Bevacizumab/Erlotinib in Non-Small Cell Lung Cancer

**DOI:** 10.1371/journal.pone.0072966

**Published:** 2013-09-10

**Authors:** Florent Baty, Sacha Rothschild, Martin Früh, Daniel Betticher, Cornelia Dröge, Richard Cathomas, Daniel Rauch, Oliver Gautschi, Lukas Bubendorf, Susanne Crowe, Francesco Zappa, Miklos Pless, Martin Brutsche, Swiss Group for Clinical Cancer Research

**Affiliations:** 1 Division of Pulmonary Medicine, Cantonal Hospital, St. Gallen, Switzerland; 2 Medical Oncology, University Hospital, Basel, Switzerland; 3 Department of Oncology, Cantonal Hospital, St. Gallen, Switzerland; 4 Department of Oncology, Cantonal Hospital, Fribourg, Switzerland; 5 Department of Oncology, Cantonal Hospital Graubünden, Chur, Switzerland; 6 Medical Oncology, Regional Hospital, Thun, Switzerland; 7 Medical Oncology, Cantonal Hospital, Luzern, Switzerland; 8 Institute of Pathology, University Hospital, Basel, Switzerland; 9 Coordinating Center, Swiss Group for Clinical Cancer Research, Bern, Switzerland; 10 Department of Medical Oncology, Oncology Institute of Southern Switzerland, Bellinzona, Switzerland; 11 Department of Oncology, Cantonal Hospital, Winterthur, Switzerland; University Magna Graecia, Italy

## Abstract

Activating epidermal growth factor receptor (EGFR) mutations are recognized biomarkers for patients with metastatic non-small cell lung cancer (NSCLC) treated with EGFR tyrosine kinase inhibitors (TKIs). EGFR TKIs can also have activity against NSCLC without EGFR mutations, requiring the identification of additional relevant biomarkers. Previous studies on tumor EGFR protein levels and EGFR gene copy number revealed inconsistent results. The aim of the study was to identify novel biomarkers of the response to TKIs in NSCLC by investigating whole genome expression at the exon-level. We used exon arrays and clinical samples from a previous trial (SAKK19/05) to investigate the expression variations at the exon-level of 3 genes potentially playing a key role in modulating treatment response: EGFR, V-Ki-ras2 Kirsten rat sarcoma viral oncogene homolog (KRAS) and vascular endothelial growth factor (VEGFA). We identified the expression of EGFR exon 18 as a new predictive marker for patients with untreated metastatic NSCLC treated with bevacizumab and erlotinib in the first line setting. The overexpression of EGFR exon 18 in tumor was significantly associated with tumor shrinkage, independently of EGFR mutation status. A similar significant association could be found in blood samples. In conclusion, exonic EGFR expression particularly in exon 18 was found to be a relevant predictive biomarker for response to bevacizumab and erlotinib. Based on these results, we propose a new model of EGFR testing in tumor and blood.

## Introduction

The prognosis of patients with stage IV non-small cell lung cancer (NSCLC) continues to be poor. Despite standard cytotoxic chemotherapy, almost 50% will not survive more than 12–14 months [1], [2]. In the past few years, improvements in survival rates have primarily been achieved by the discovery of predictive molecular markers which identified subgroups of patients deriving a substantial benefit from targeted treatment. Several randomized phase III trials have recently shown a significant benefit of epidermal growth factor receptor tyrosine kinase inhibitors (EGFR-TKIs) in chemotherapy naïve patients harboring an activating EGFR mutation [3]–[6]. EGFR mutations are found in about 10–15% of Caucasian patients [7]. In EGFR wild-type patients the first-line treatment with an EGFR-TKI might even harm compared to conventional chemotherapy [8]. However, in unselected chemotherapy-naïve patients the role of EGFR-TKIs is less clear and previous studies have demonstrated inferior outcomes with TKIs with or without bevacizumab compared to chemotherapy [9]–[11]. These results indicate, that there is a subgroup of EGFR wild-type patients who might benefit from treatment with a TKI or a TKI plus an anti-angiogenic agent. The same holds true for unselected and pretreated patients where the role of TKIs has been addressed in numerous trials and the efficacy and survival rates have shown to be comparable to conventional chemotherapy [12]–[14]. Furthermore, recent biomarker analyses of three large trials testing maintenance therapy with erlotinib clearly demonstrated, that a subset of EGFR wild-type patients also derive a significant benefit from EGFR-TKI therapy [15]–[17].

Beside EGFR other druggable oncogenic mutations in advanced NSCLC have been described [18], [19]. Unfortunately, most patients with NSCLC do not harbor a corresponding molecular target hence chemotherapy continues to be their first treatment of choice. Therefore, the identification of further subgroups of patients who may derive benefit from targeted treatment by exploring additional molecular markers is crucial.

Treatment with bevacizumab and erlotinib (BE) has potential benefits over chemotherapy, particularly in regard to its more favorable toxicity profile. There is evidence, that the addition of the vascular endothelial growth factor (VEGF) targeting monoclonal antibody bevacizumab to the EGFR-TKI erlotinib exhibits increased efficacy compared with erlotinib alone in unselected patients who were previously treated with chemotherapy [20]. This observation likely results from enhanced erlotinib activity, given the lack of efficacy of bevacizumab monotherapy in lung cancer.

The Swiss Group for Clinical Cancer Research (SAKK) recently reported a median time to progression (TTP) of 4.1 months in patients with untreated advanced non-squamous NSCLC treated with BE [21]. This result appears to be inferior to what would be expected with modern chemotherapy combinations in similar patient populations [2], [22]. In the current substudy, we aimed to identify a potential subgroup of patients participating in the SAKK 19/05 trial, particularly within the EGFR wild-type group, who may benefit from treatment with BE. The main goal of this study was to assess the correlation of exon-level expression variations of 3 specific genes [EGFR, V-Ki-ras2 Kirsten rat sarcoma viral oncogene homolog (KRAS) and vascular endothelial growth factor A (VEGFA)] and the response to first line BE therapy in patients who participated in the SAKK 19/05 trial.

## Results

### Patient characteristics and clinical outcome

The SAKK 19/05 trial included 103 patients, 101 were evaluable for the primary statistical analysis. Overall, median age was 65 (range, 32–80) years. All patients were in a good performance status (WHO 0-1), 48 were male (48%), 53 were female (52%). The majority (86%) had stage IV disease. EGFR mutations were identified in 15 patients (15%). One patient had a primary resistance mutation T790M in exon 20. KRAS mutation were identified in 13 patients (13%). Objective tumor responses at 12 weeks (PR or CR) were observed in 15 patients (15%). These patients had the following EGFR mutational status: EGFR del19 (n = 5), L858R (n = 2), unknown mutational status (n = 1), and EGFR wild-type (n = 8). One patient with EGFR wild-type and response to BE therapy had a KRAS mutation G12D.

From these patients, tumor tissue for exon array analysis was obtained from 42 patients and blood samples from 75 patients (Table S1 in the Supporting Information). A detailed description of patient characteristics is provided in Table 1 (tumor tissue samples) and in Table 2 (blood samples). Tissue samples corresponded to our primary dataset used for biomarker identification. Blood samples were used for confirmatory purpose (validation set).

**Table 1:** Patients' details for patients with treatment naive biopsies.

UPN	Age	Gender	Stage	Smoking status	DST W12	EGFR mut (18–21)	KRAS mut (12)	Tumor shrinkage W12 (%)
2	69	M	IV	smoker	0	NA	NA	65
23	53	F	IV	smoker	0	no	no	17
38	58	F	IV	never smoker	0	NA	NA	NA
49	56	M	IV	smoker	1	no	no	−15
51	70	F	IIIB	never smoker	0	no	no	18
55	55	F	IV	smoker	0	no	no	NA
56	61	F	IV	never smoker	1	NA	NA	23
57	66	F	IV	smoker	0	no	no	NA
58	46	F	IV	smoker	0	no	G12D	NA
60	64	F	IV	never smoker	1	no	G12D	53
61	61	F	IV	never smoker	1	L858R	no	36
63	48	F	IIIB	smoker	0	no	no	NA
64	64	M	IV	smoker	1	no	no	−1
65	67	F	IV	never smoker	0	no	no	NA
67	53	M	IV	smoker	1	no	no	−1
68	63	M	IV	smoker	0	no	G12D	NA
69	66	F	IIIB	smoker	0	no	no	−2
70	35	M	IV	smoker	1	no	no	5
74	61	M	IV	never smoker	1	no	no	66
75	61	M	IV	never smoker	0	no	no	NA
76	51	F	IV	smoker	1	no	G12C	3
77	54	M	IV	smoker	1	no	no	16
78	63	F	IV	smoker	1	Del L747-G749	NA	26
80	44	F	IV	smoker	0	NA	NA	NA
81	55	M	IV	smoker	0	no	no	NA
82	58	M	IV	smoker	1	no	no	0
83	53	F	IV	smoker	0	no	G12D	NA
84	55	F	IV	never smoker	1	NA	NA	0
87	74	M	IV	smoker	1	no	no	−15
88	78	M	IV	never smoker	0	no	no	−3
90	69	F	IV	smoker	1	no	no	0
91	68	M	IV	smoker	0	no	no	NA
93	56	F	IV	never smoker	1	E709A and G719S	no	12
94	49	F	IV	smoker	1	no	G12V	16
95	64	M	IV	smoker	1	NA	NA	1
96	77	M	IV	smoker	0	no	no	NA
97	68	F	IV	smoker	0	no	no	NA
98	64	F	IV	never smoker	1	no	no	18
99	48	M	IV	smoker	1	no	no	−6
101	66	M	IV	smoker	0	no	no	−1
102	59	F	IV	smoker	1	no	no	−8
103	72	F	IV	never smoker	1	Del E746-A750	no	76

Abbreviations: DST W12: disease stabilization week 12, 0 = failure, 1 = success; EGFR mut (18–21): EGFR mutation in exons 18–21; KRAS mut (12): KRAS mutation in exon 12; W12: week 12.

**Table 2:** Patients' details for patients in the blood study.

UPN	Age	Gender	Stage	Smoking status	DST W12	EGFR mut (18–21)	KRAS mut (12)	Tumor shrinkage W12 (%)
2	69	M	IV	smoker	0	NA	NA	65
3	50	M	IV	smoker	1	no	G12C	−6
7	55	M	IV	smoker	0	no	G12C	−33
8	65	F	IV	smoker	0	no	no	NA
9	61	M	IV	smoker	0	no	no	−41
14	53	M	IV	smoker	0	no	NA	15
15	55	F	IV	smoker	0	no	NA	NA
16	75	M	IV	never smoker	1	Del L747-E749	no	100
18	64	M	IV	never smoker	1	L858R	no	45
19	62	M	IV	smoker	0	NA	NA	NA
20	74	M	IV	smoker	1	no	no	6
21	59	F	IV	smoker	1	no	no	43
23	53	F	IV	smoker	0	no	no	17
25	59	M	IV	smoker	0	no	no	NA
26	58	M	IV	smoker	1	no	no	11
27	72	M	IV	smoker	0	NA	NA	−22
28	68	F	IV	smoker	0	no	no	NA
29	57	M	IIIB	smoker	1	no	no	13
30	65	F	IIIB	never smoker	0	no	no	NA
37	61	M	IV	smoker	0	no	NA	NA
38	58	F	IV	never smoker	0	NA	NA	NA
39	68	M	IV	smoker	0	R705GA	G12A	NA
40	53	M	IV	smoker	1	no	no	5
42	51	F	IV	smoker	1	no	no	23
44	51	F	IV	smoker	0	no	G12C	NA
47	72	M	IV	smoker	1	no	no	25
49	56	M	IV	smoker	1	no	no	−15
50	63	M	IV	smoker	1	NA	NA	12
51	70	F	IIIB	never smoker	0	no	no	18
53	49	F	IV	smoker	1	NA	NA	−12
54	49	M	IV	smoker	1	Del L747-S751_InsS	no	27
55	55	F	IV	smoker	0	no	no	NA
56	61	F	IV	never smoker	1	NA	NA	23
57	66	F	IV	smoker	0	no	no	NA
58	46	F	IV	smoker	0	no	G12D	NA
59	47	F	IV	smoker	1	no	no	−7
60	64	F	IV	never smoker	1	no	G12D	53
61	61	F	IV	never smoker	1	L858R	no	36
63	48	F	IIIB	smoker	0	no	no	NA
64	64	M	IV	smoker	1	no	no	−1
65	67	F	IV	never smoker	0	no	no	NA
66	57	F	IIIB	never smoker	1	no	no	12
67	53	M	IV	smoker	1	no	no	−1
68	63	M	IV	smoker	0	no	G12D	NA
69	66	F	IIIB	smoker	0	no	no	−2
70	35	M	IV	smoker	1	no	no	5
72	56	F	IIIB	smoker	1	no	G12D	−15
73	54	M	IV	smoker	0	no	NA	NA
74	61	M	IV	never smoker	1	no	no	66
75	61	M	IV	never smoker	0	no	no	NA
77	54	M	IV	smoker	1	no	no	16
78	63	F	IV	smoker	1	Del L747-G749	NA	26
79	32	F	IIIB	never smoker	1	Del E746-A750	no	100
80	44	F	IV	smoker	0	NA	NA	no NA
81	55	M	IV	smoker	0	no	no	NA
82	58	M	IV	smoker	1	no	no	0
83	53	F	IV	smoker	0	no	G12D	NA
84	55	F	IV	never smoker	1	NA	NA	0
85	48	F	IV	smoker	1	no	no	0
86	56	F	IV	smoker	0	NA	NA	NA
87	74	M	IV	smoker	1	no	no	−15
88	78	M	IV	never smoker	0	no	no	−3
89	69	F	IV	never smoker	1	Del R748-S752	no	62
90	69	F	IV	smoker	1	no	no	0
91	68	M	IV	smoker	0	no	no	NA
92	64	F	IV	never smoker	1	Del E746-A750	no	39
93	56	F	IV	never smoker	1	E709A and G719S	no	12
94	49	F	IV	smoker	1	no	G12V	16
95	64	M	IV	smoker	1	NA	NA	1
96	77	M	IV	smoker	0	no	no	NA
98	64	F	IV	never smoker	1	no	no	18
99	48	M	IV	smoker	1	no	no	−6
101	66	M	IV	smoker	0	no	no	−1
102	59	F	IV	smoker	1	no	no	−8
103	72	F	IV	never smoker	1	Del E746-A750	no	76

Abbreviations: DST W12: disease stabilization week 12, 0 = failure, 1 = success; EGFR mut (18–21): EGFR mutation in exons 18–21; KRAS mut (12): KRAS mutation in exon 12; W12: week 12.

### Target gene expression analysis on exon-level

#### Epidermal growth factor-receptor (EGFR)

**Figure 1:**
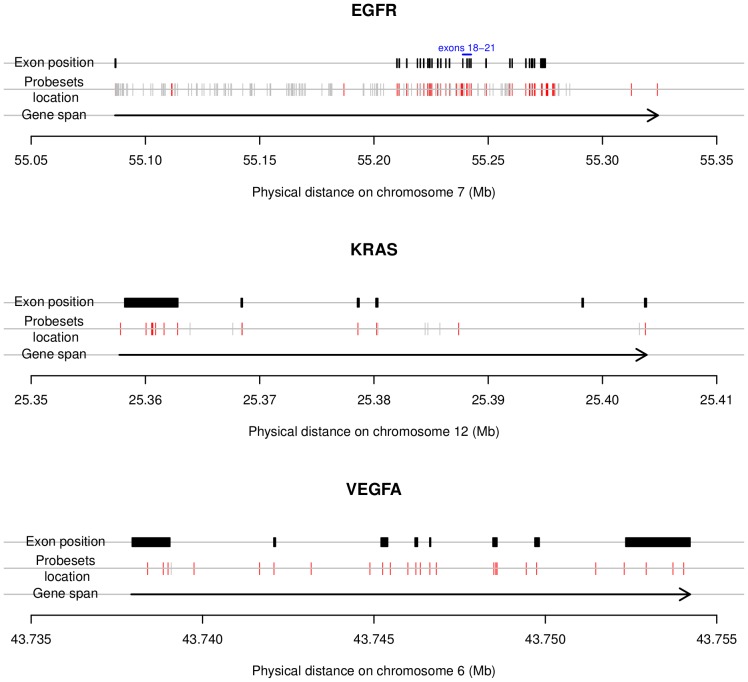
Chromosomal location of the Affymetrix exon array probesets within EGFR, KRAS and VEGFA. The red ticks show the exonic probesets, the gray ticks display the non-exonic probesets (intronic, intergenic and unreliable). In EGFR, KRAS and VEGFA, a total of 51 of 451, 13 of 262 and 25 of 26 exonic probesets were measured respectively. All other probesets were situated outside of exons, *i.e.* intronic, intergenic or were unreliable.

EGFR gene expression was measured at 451 loci, of which 51 were situated within exons, and 400 were situated outside of exons, *i.e.* intronic, intergenic or were unreliable (Figure 1, upper panel). Thus, a total of 51 exon probesets expression intensities were measured within the EGFR gene. A summary measure of all these exon-level probesets was provided by PCA (scores on the first PC axis). The association between this score and TS12 and TTP under BE, OS, and TTP under chemotherapy was evaluated.

**Figure 2:**
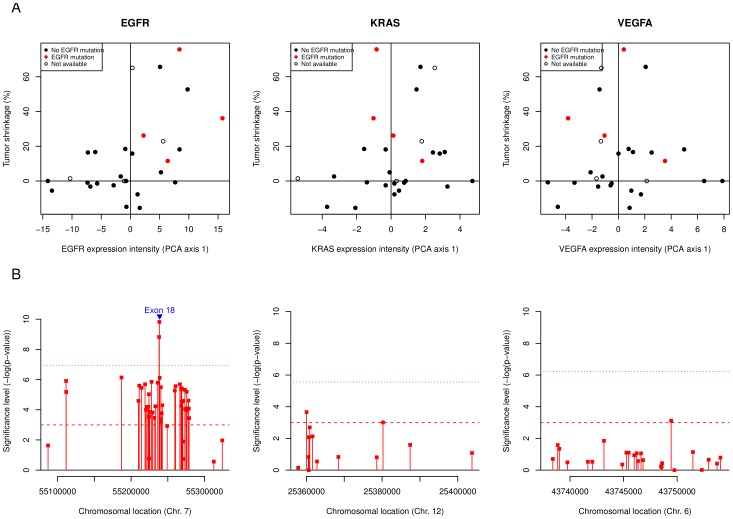
Association between EGFR, KRAS and VEGFA exon-level expression and response to BE. Row A depicts the association between the tumor shrinkage at week 12 and the exon-level composite score (PCA axis 1) for EGFR, KRAS and VEGFA (left, center and right respectively). The PCA scores are defined as the coordinates of the patients in a new space defined by linear combination of the original probeset intensity values using principal component analysis. The patients with EGFR mutations are marked in red, those with non-available mutational status are shown as empty circles. The row B shows the significance of the correlation (−log(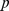-value)) between each exon probeset and the tumor shrinkage at week 12. The position of the exons is shown in blue.

We found a significant correlation between EGFR PCA scores and TS12 after BE treatment (Spearman's 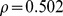, 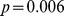) (Figure 2A, left panel). A detailed analysis probeset-by-probeset revealed that 86% of the exonic probesets showed a significant correlation with tumor shrinkage without correction for multiple testing (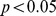) (Figure 2B, left panel). Two probesets showed a particularly strong correlation with TS12 (exon probesets ID 3002770 and 3002769), which remained significant after Bonferroni correction for multiple testing. These 2 probesets are located on exon 18 (chromosome 7, positions 55'238'440 and 55'238'092, respectively). No other significant associations were found. Six patients had TTP of 15 months or more. Three of those had EGFR del19, and 3 were EGFR and KRAS wild-type.

**Figure 3:**
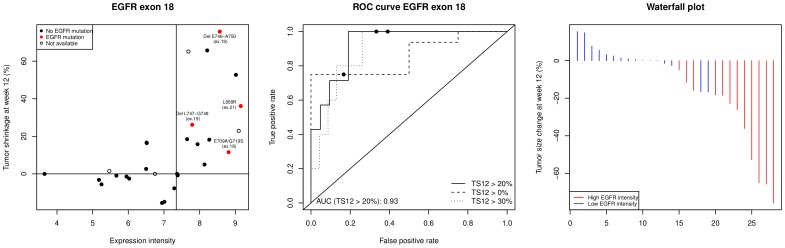
Exon 18-EGFR expression is associated with tumor shrinkage. The left panel depicts the correlation between the expression intensity of the exon 18-EGFR (probeset 3002770) and the tumor shrinkage at week 12. The vertical line shows the median expression intensity of EGFR probeset 3002770. Patients with EGFR mutations are shown as red plain dots and labelled accrodingly. Patients with non-available mutational status are displayed as empty circles. The central panel represents the receiver operating characteristic (ROC) curve showing the sensitivity/specificity of a test based on the expression level of EGFR probeset 3002770 to classify responders (tumor shrinkage at week 12
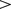
0/20/30%) vs. non-responders (tumor shrinkage at week 12
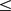
0/20/30%). The plain dots depict the true positive and false positive rates obtained by fixing the cutoff value to the median expression level of EGFR 3002770. The waterfall plot (right panel) displays the change in tumor size at week 12 ordered from left to right. The colors are defined by the expression intensity of EGFR 3002770 dichotomized by the median of the expression evel (blue: low expression intensities; red: high expression intensities).

Figure 3 depicts the significant association of exon 18-EGFR expression intensity and TS12. The left panel shows a strong association between the expression intensity of exon 18-EGFR (probeset 3002770) and TS12 (Spearman's 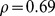, 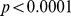).

The strong correlation between EGFR exon 18 expression and TS12 remained highly significant (Spearman's 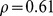, 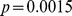) after restricting the analysis to EGFR wild type patients (see Figure S1 in the supporting information). This sub-analysis indicates that the association between EGFR exon 18 expression and TS12 was independent from the EGFR mutation status.

The ROC analysis (middle panel) shows the relationship between sensitivity and specificity depending on different cut-off levels of exon 18-EGFR (probeset 3002770) expression to classify patients into “responders” vs. “non-responders”. For the purpose of this ROC analysis, the categorization “responders” vs. “non-responders” derived from TS12. We proposed 3 alternative definitions to “responders” by setting the TS12 cut-off as greater or equals to 0, 20, or 30%, depending on whether or not one included all or a fraction of stable disease patients in the “responders” category. Using the median expression of EGFR probeset 3002770 as test-threshold provides a classification accuracy of 75% (sensitivity = 100%, specificity = 67%). As shown in the ROC curve, a higher classification accuracy can be expected by further fine tuning this threshold (area under curve [AUC] = 0.93).

The 2 exon 18-EGFR probesets showing the strongest correlation with TS12 also showed a significant association for the same endpoint when measured using blood (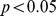).

The stability of our finding was assessed using bootstrapping, and cross-validation strategies. The procedure confirmed the strong classification accuracy of exon 18 EGFR with a median ROC-AUC of 0.94 (95% CI: 0.70–1.00) and the specific association between the exon 18 region and tumor shrinkage at week 12 (see Figure S2 and Text S1 for detailed procedure).

#### Kirsten rat sarcoma viral oncogene homolog (KRAS) and vascular endothelial growth factor-alpha (VEGFA)

In total, 13 and 25 exon probesets expression intensities were measured within KRAS and VEGFA respectively (Figure 1, central and right panels). The PCA scores obtained for both sets of probeset (KRAS and VEGFA) did not show significant association with any of the clinical endpoints. A detailed analysis probeset-by-probeset did not reveal any significant association with either TS12 (Figure 2A, B, central and right panels) or the other investigated endpoints.

## Discussion

To our knowledge, this is the first study exploring the correlation between gene expression assessed at a subgenic exonic level using Affymetrix Human Exon 1.0 ST arrays and response to treatment with an EGFR-TKI in combination with an anti-angiogenic agent. We investigated the exon intensity variations within 3 key genes (EGFR, KRAS and VEGFA) potentially associated with response to treatment with BE. We were able to demonstrate a strong association between the majority, but not all, of the 51 EGFR exon probesets and TS12 of first-line BE therapy in patients with untreated advanced non-squamous NSCLC. Exon 18-EGFR levels showed the best association with response to BE. Based on our previous experiments we assume that the signal we measured in EGFR Exon 18 did not depend on the tumor cell content [23]. Furthermore, there was a quantitative relationship - higher EGFR mRNA level was correlated with more pronounced tumor shrinkage, independently of EGFR mutational status. EGFR exon-level expression analysis might become a useful biomarker for daily clinical practice as it provides several advantages in comparison to conventional mutational analysis by gene sequencing. Typically, EGFR gene expression is measured using quantitative RT-PCR with primers binding to a single gene region often near the 3′-end of the gene. However, as shown in our study, gene expression did significantly vary over the span of the EGFR gene. Reasons for such expression variations include alternative splicing. The EGFR variant type III (EGFRvIII) has an in-frame deletion of exons 2–7 which has been found to be generated by gene rearrangement or aberrant mRNA splicing [24], [25]. This alternative splicing form has been found in NSCLC [26], [27]. In preclinical experiments, cells expressing EGFRvIII were resistant against reversible EGFR-TKIs, but remained sensitive to irreversible EGFR inhibitors [28]. We found the best correlation with TS12 and exon 18. At the extremities of the EGFR gene several exonic probesets did not show a significant association with outcome. Dziadziuszko and colleagues reported that high EGFR mRNA expression analyzed by quantitative RT-PCR was associated with increased response and prolonged PFS in patients treated with gefitinib [29]. In a Chinese study of 79 unselected patients treated with erlotinib no significant correlation between EGFR mRNA expression, EGFR mutations, KRAS mutations and clinical endpoints was found [30].

Several trials demonstrated that clinical benefit with EGFR-TKIs was not restricted to patients with activating EGFR mutations [13], [16], [31]. On the other hand, the IPASS trial demonstrated that patients with EGFR wild-type treated with gefitinib had a significantly shorter PFS compared with patients in the chemotherapy arm (hazard ratio (HR): 2.85; 95% CI: 2.05–3.98; 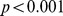) [8]. In the present study, we were able to identify 3 patients with EGFR wild-type and high exon 18-EGFR expression levels (2 measured in biopsies and blood, and 1 measured in blood only) who had significant TS12 after treatment with BE. We believe that these results are of interest, because the incidence of activating EGFR mutations in Caucasian patients is 10–15% and our test may identify additional patients who could fare better with first-line EGFR-TKIs compared with chemotherapy. This hypothesis needs prospective validation.

Interestingly, patients with rarer EGFR-mutations (*e.g.* del L747-S751 and del R748-S752) for which the response to EGFR-TKIs has yet to be explored were also found to have higher exon-level EGFR expression levels which was correlated with TS12. Two probesets located on exon 18 showed the strongest association with tumor shrinkage. In an Italian single institution study, rare EGFR-mutations (exon 18 and 20 and uncommon mutations in exons 19 and 21 and/or complex mutations) were found in 2.6% of patients. They reported PR to erlotinib in a patient with a E709A+G719C double mutation and a response to erlotinib in a patient with a G719S mutation [32]. Other groups reported sensitivity to EGFR-TKI for the E709A+G719C double mutation and for the G719S mutation in exon 18 [33]–[35].

Interestingly, we observed tumor shrinkage in one patient with a KRAS mutation. This patient had a high EGFR exon expression. Patients with KRAS mutations represent approximately 25% of NSCLC patients and have been described as highly resistant to EGFR-TKI treatment with RR close to 0% and worse outcome for mutated patients treated with EGFR-TKIs in some trials [36], [37]. The biomarker analysis of the SATURN trial showed no detrimental effect on PFS with erlotinib in patients with KRAS mutant tumors [17]. Thus, high exon EGFR expression levels may be able to identify patients with KRAS mutations who derive benefit from first-line BE.

Other potential molecular markers beyond EGFR-mutations have been investigated for their predictive role for treatment with TKIs or TKIs in combination with VEGFR inhibitors. EGFR protein expression detected by immunohistochemistry (IHC) is present in 60–90% of NSCLC patients [13], [38] and therefore unlikely to be of use for clinical selection for TKI therapy. Although subgroup analyses of placebo controlled phase III studies in pre-treated patients showed some predictive value of EGFR protein expression [13], [39], these results were not confirmed either in the first line or maintenance setting [17], [40]. Similarly, high EGFR copy number, which occurs in 30–50% of patients with NSCLC, and gene amplification, which occurs in about 10% [41], have recently been shown to be €overruled€ by EGFR mutations with respect to their predictive value for the response to EGFR-TKIs [40]. Determination of EGFR mRNA expression by quantitative PCR was correlated to EGFR FISH and IHC and was shown to be a predictive biomarker for gefitinib [29]. Neither EGFR protein expression nor EGFR FISH testing are currently used in clinical practice and better molecular markers are therefore urgently needed.

The EGFR gene gives rise to multiple RNA transcripts through alternative splicing and the use of alternate polyadenylation signals [42]. The EGFR gene spans nearly 200 kb and the full-length 170 kDa EGFR is encoded by 28 exons. Several alternative splicing variants have been described [43]. The most commonly used method to detect EGFR-mutations is direct sequencing of the PCR-amplified exon sequences. The copy number of mutant allele, imbalanced PCR amplification and the relative amount of contaminating wild-type allele of non-tumor cells can influence the sensitivity of mutant detection by direct sequencing [44]. Owing to concern regarding the sensitivity of the direct-sequencing method, a variety of other methods have been investigated to increase the sensitivity of the mutation assay. Here we investigated for the first time exon expression analysis. The array used enables gene expression analysis as well as detection of different isoforms of a gene. In this study we retrospectively identified a correlation between exon intensity levels within EGFR and patient outcome. The mechanism through which EGFR exon 18 expression determines an increased sensitivity to bevacizumab-erlotinib is unknown, although different hypotheses can be proposed. Exon array is still very recent with high potential technology. It brakes with the common idea that gene expression is stable over the span of a whole gene. Therefore, it is not surprising that we obtained a stronger statistical correlation EGFR expression near the region coding for the functional transmembrane part of EGFR. If the predictive value of this assay could be confirmed in a prospective trial, exon-level gene expression might identify patients deriving benefit from EGFR- and VEGFR-targeted therapies beyond the patients selected by conventional gene sequencing.

There are certain limitations within the current study. It is a single arm design and has a relatively low number of patients from which tumor biopsies were available for analysis. In the first half of the SAKK 19/05 trial a treatment-naive biopsy was not required for study inclusion. In this period practically no biopsies were collected. After an amendment (October 2006) the biopsy became mandatory for study inclusion as a treatment-naive biopsy can be taken in almost every patient including advanced-stage NSCLC patients [23]. Exon array analyses were done with mixed cell tumor biopsies without any tumor-cell enriching technique like laser-capture microdissection. This is likely to lead to a certain dilution of the true tumor signal. Tumor-cell enriching techniques might further optimize the efficiency of biomarkers derived from exon array analyses. The validity of EGFR exon expression analysis as a biomarker of response to BE will need to be confirmed both using RT-PCR analysis targeting EGFR exon 18. The full accomplishment of the validation of the novel biomarker eventually requires further investigation using an independent prospective randomized trial.

In conclusion, with the aid of a novel gene expression array technology with exonic coverage, we were able to identify exon 18-EGFR expression as a potential predictive biomarker for erlotinib and bevacizumab in patients with advanced, untreated NSCLC.

## Materials and Methods

### SAKK 19/05

The SAKK 19/05 trial (ClinicalTrials.gov: NCT00354549) enrolled 103 patients with advanced non-squamous NSCLC, 101 patients were evaluable for further analysis [21]. Eligibility criteria included age 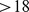 years, adequate bone marrow function, normal kidney and liver function and measurable disease. Patients with immediate need of chemotherapy, with large centrally located tumors, pre-existing tumor cavitations and brain metastases were excluded. Extra pre-treatment bronchoscopic biopsies for translational studies were taken in 49 patients, from which 42 were of sufficient quality for subsequent exon array analysis. For the present substudy, pretreatment blood samples were available from 95 patients, and samples from 75 patients had sufficient quality for exon arrays. Overall, 76 patients with either tumor or blood samples or both, were included in the current substudy. Written informed consent for translational research was obtained from all patients. The clinical trial as well as the current substudy were approved by the IRB of St. Gallen (EKSG 06/012).

### Trial design

SAKK 19/05 was a multicenter, prospective, open-label, single-arm, phase II trial in previously untreated patients. From January 2006 to April 2009, 103 patients from 14 Swiss institutions were enrolled and received BE until disease progression or unacceptable toxicity. At the time of progression, patients received chemotherapy with 4–6 cycles of cisplatin and gemcitabine. The primary endpoint was disease stabilization rate (DSR) defined as the proportion of patients with complete response (CR), partial remission (PR) or stable disease (SD) after 12 weeks of BE treatment. Secondary endpoints included TTP under BE, as well as under CT, overall survival (OS), tumor shrinkage at 12 weeks and 6 months. The clinical outcomes of this trial have been reported earlier [21].

### Pathology analysis

The formalin-fixed and paraffin embedded specimens were reviewed and classified according to World Health Organisation (WHO) criteria. Mutational analyses of EGFR (exon 18–21) and KRAS (exon 12) were carried out from unstained tissue sections (3 µm) or Papanicolaou-stained cytological specimens using direct sequencing as previously described [45], [46]. Tumor cell enrichment was achieved either by macrodissection or laser-capture microdissection and DNA sequence analysis.

### Exon-level gene expression analysis

Total RNA from whole bronchoscopic biopsy samples were extracted and provided sufficient quality for microarray hybridization in 42 of 49 samples. Circulating RNA from peripheral blood samples was extracted and provided sufficient quality for microarray hybridization in all 75 samples. mRNA was hybridized on Affymetrix Human Exon 1.0ST arrays (Affymetrix, SantaClara, CA, USA) following standard recommendations from the manufacturer (detailed procedure available in Text S1). Raw data have been deposited in NCBIs Gene Expression Omnibus (GEO), and are accessible through GEO Series accession number GSE37138. The exon and gene level probesets were pre-processed, quality checked and normalized using the RMA procedure [47]. The tissue and blood datasets were analyzed independently without pooling the data. The tissue dataset was used for biomarker discovery whereas the blood dataset was used for internal validation.

### Statistical considerations

The initial sample size calculation was based on the primary endpoint of the clinical study (DSR at week 12 (DSR12) under BE treatment). The 101 evaluable patients accrued guaranteed a high precision in the estimation of DSR12. In a targeted gene approach, 3 genes were specifically investigated: EGFR (ENSG00000146648), KRAS (ENSG00000133703) and VEGFA (ENSG00000112715). EGFR included 51, KRAS 13, and VEGFA 25 exonic probesets (Figure 1). The endpoints considered in this biomarker study included tumor shrinkage after 12 weeks (TS12) of BE treatment, TTP under BE and OS. OS was measured from registration until death of any cause. The result of previous tumor EGFR sequencing was used for substudy analysis. The univariate association between the exon-level intensities and time-to-event endpoints was assessed by Cox proportional hazards regression. The correlation between exon-level intensities and tumor shrinkage was measured using the Spearman's correlation coefficient 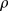 and tested for significant difference from 0. Bonferroni corrections were used to account for multiple testing. Principal component analysis (PCA) was used to summarize the information included in several exon-level probesets into composite scores (scores on the first principal components). Receiver Operating Characteristic (ROC) curves were used to estimate the sensitivity, specificity and accuracy of exon expression based predictors. In order to assess the stability of our findings, a cross-validation strategy was used. The accuracy of the classification model was evaluated using bootstrapping. All analyses were done using the R statistical software (version 2.13.0; packages xmapcore, ade4, ROCR, Daim and survival) [48].

## Supporting Information

Figure S1 **Association between EGFR exon 18 expression and tumor shrinkage at week 12 — sub-analysis.** Only EGFR wild type patients were included in this analysis. The scatter plot depicts the correlation between the expression of EGFR exon 18 (probeset 3002770) and the tumor shrinkage at week 12. The vertical line shows the median expression intensity of EGFR exon 18.
(TIF)

Figure S2 **Stability of the prediction ability of EGFR biomarkers using cross-validation strategies.** The left panel depicts the ability of the EGFR biomarker most significantly associated with TS12 (≤/>20%) using the original dataset (probeset 3002770) to classify BE responders. The best cut-off value, together with the associated false positive rate (FPR), true positive rate (TPR) and area under ROC curve (AUC) are given. The right panel depicts the averaged ROC curve obtained after .632 bootstrap cross-validation procedure. The boxplots show the distribution of the FPR throughout the re-sampled datasets.
(TIF)

Table S1 **Summary of all patients included in the SAKK 19/05 trial.** DST W12: disease stabilization week 12, 0 = failure, 1 = success.
(PDF)

Text S1 **Additional material and methods information.** The first paragraph provides an extended description of the exon-level gene expression analysis. The second paragraph gives details about the assessment of the stability of the obtained results.
(PDF)
