# Supplementary figures and images for: EGFR Exon-Level Biomarkers of the Response to Bevacizumab/Erlotinib in Non-Small Cell Lung Cancer
Source: PLoS One. 2013 Sep 10;8(9):e72966. doi: 10.1371/journal.pone.0072966 (PMC3769372; doi:10.1371/journal.pone.0072966)

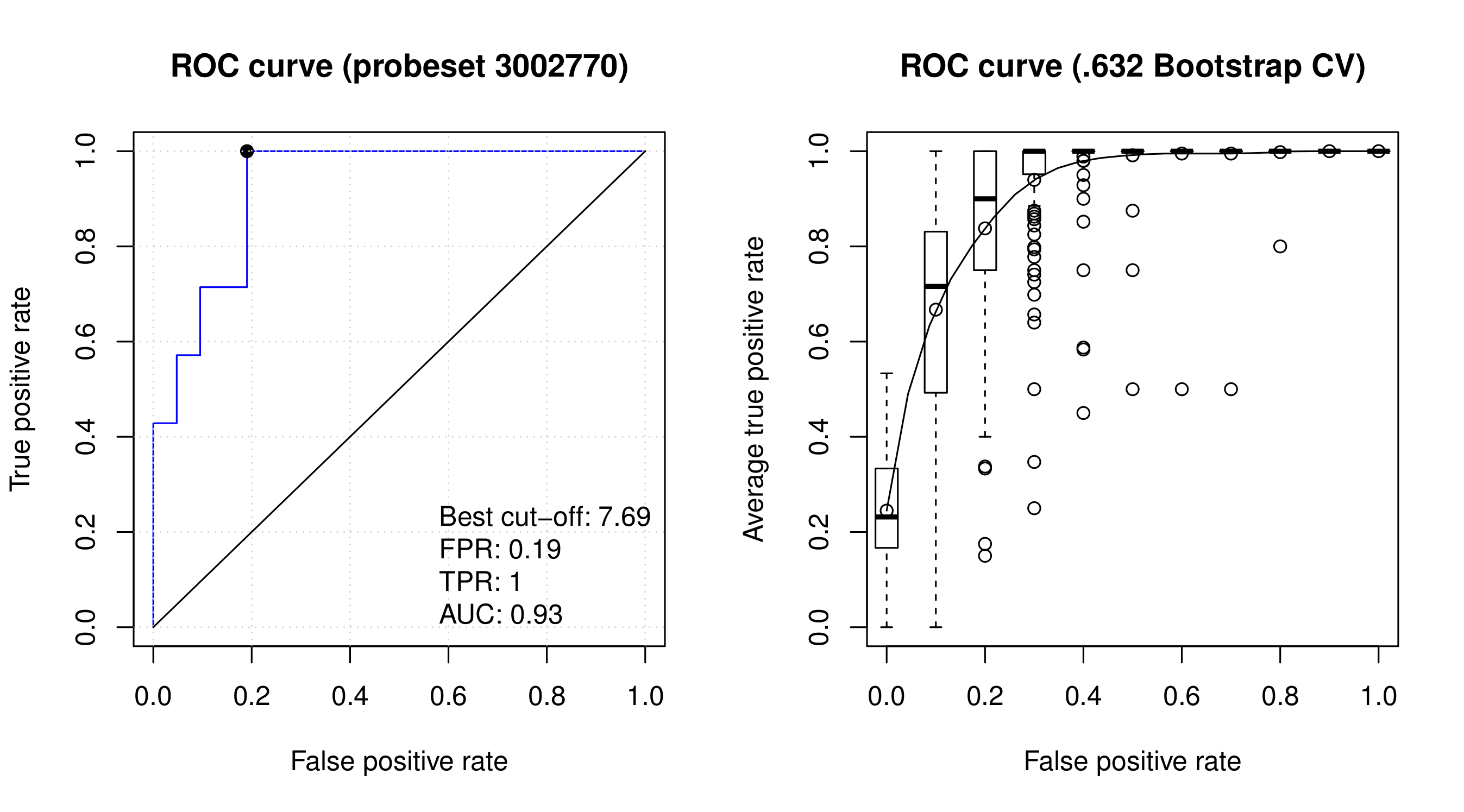

Supplement: Figure S2 — Stability of the prediction ability of EGFR biomarkers using cross-validation strategies. The left panel depicts the ability of the EGFR biomarker most significantly associated with TS12 (≤/>20%) using the original dataset (probeset 3002770) to classify BE responders. The best cut-off value, together with the associated false positive rate (FPR), true positive rate (TPR) and area under ROC curve (AUC) are given. The right panel depicts the averaged ROC curve obtained after .632 bootstrap cross-validation procedure. The boxplots show the distribution of the FPR throughout the re-sampled datasets. (TIF) [file pone.0072966.s002.tif]
