# Supplementary material for: EGFR Exon-Level Biomarkers of the Response to Bevacizumab/Erlotinib in Non-Small Cell Lung Cancer
Source: PLoS One. 2013 Sep 10;8(9):e72966. doi: 10.1371/journal.pone.0072966 (PMC3769372; doi:10.1371/journal.pone.0072966)
Supplement: Table S1 — Summary of all patients included in the SAKK 19/05 trial. DST W12: disease stabilization week 12, 0 = failure, 1 = success. (PDF) [file pone.0072966.s003.pdf]

UPN	Age	Gender	Disease stage	DST W12	Objective response under BE	EGFR mutation	Biopsy study	Blood study
1	70	F	IV	0	no	—	—	—
2	69	M	IV	0	yes	—	×	×
3	50	M	IV	1	no	—	—	×
4	49	M	IV	0	no	—	—	—
5	61	F	IV	1	no	—	—	—
6	61	F	IV	1	yes	—	—	—
7	55	M	IV	0	no	—	—	×
8	65	F	IV	0	no	—	—	×
9	61	M	IV	0	no	—	—	×
10	59	M	IV	1	no	—	—	—
11	77	M	IIIB	1	no	—	—	—
12	70	M	IIIB	1	no	—	—	—
13	55	M	IV	0	no	—	—	—
14	53	M	IV	1	no	—	—	×
15	55	F	IV	0	no	—	—	×
16	75	M	IV	1	yes	del(19)	—	×
17	77	F	IIIB	1	yes	del(19)	—	—
18	64	M	IV	1	yes	L858R(21)	—	×
19	62	M	IV	0	no	—	—	×
20	74	M	IV	1	no	—	—	×
21	59	F	IV	1	yes	—	—	×
22	50	M	IV	0	no	—	—	—
23	53	F	IV	1	no	—	×	×
24	80	F	IV	0	no	L858R(21)	—	—
25	59	M	IV	0	no	—	—	×
26	58	M	IV	1	no	—	—	×
27	72	F	IV	0	no	—	—	×
28	68	F	IV	0	yes	—	—	×
29	57	M	IIIB	1	no	—	—	×
30	65	F	IIIB	0	no	—	—	×
31	66	F	IIIB	0	yes	—	—	—
32	71	F	IV	1	no	—	—	—
33	54	F	IIIB	1	no	—	—	—
34	65	F	IV	0	no	—	—	—
36	64	M	IV	0	no	—	—	—
37	61	M	IV	0	no	del(19)	—	×
38	58	F	IV	0	no	—	×	×
39	68	M	IV	0	no	R705G(18)	—	×
40	53	M	IV	1	no	—	—	×
41	72	M	IV	0	no	—	—	—
42	51	F	IV	1	no	—	—	×
43	77	F	IV	0	no	T790(20)	—	—
44	51	F	IV	0	no	—	—	×
45	68	M	IV	1	no	—	—	—
46	63	F	IIIB	1	yes	—	—	—
47	72	M	IV	1	no	—	—	×
48	53	F	IV	0	no	—	—	—
49	56	M	IV	1	no	—	×	×
50	63	M	IV	1	no	—	—	×
51	70	F	IIIB	0	no	—	×	×
52	71	M	IV	0	no	—	—	—
53	49	F	IV	1	no	—	—	×
54	49	M	IV	1	yes	del(19)	—	×
55	55	F	IV	0	no	—	×	×
56	61	F	IV	1	no	—	×	×
57	66	F	IV	0	no	—	×	×
58	46	F	IV	0	no	—	×	×
59	47	F	IV	1	no	—	—	×
60	64	F	IV	1	NA	—	×	×
61	61	F	IV	1	yes	L858R(21)	×	×
63	48	F	IIIB	0	no	—	×	×
64	64	M	IV	1	no	—	×	×
65	67	F	IV	0	no	—	×	×
66	57	F	IIIB	1	no	—	—	×
67	53	M	IV	1	no	—	×	×
68	63	M	IV	0	yes	—	×	×
69	66	F	IIIB	0	no	—	×	×
70	35	M	IV	1	no	—	×	×
71	60	M	IV	0	no	—	—	—
72	56	F	IIIB	1	no	—	—	×
73	54	M	IV	0	no	—	—	×
74	61	M	IV	1	yes	—	×	×
75	61	M	IV	0	no	—	×	×
76	51	F	IV	1	no	—	×	—
77	54	M	IV	1	no	—	×	×
78	63	F	IV	1	no	del(19)	×	×
79	32	F	IIIB	1	yes	del(19)	—	×
80	44	F	IV	0	no	—	×	×
81	55	M	IV	0	no	—	×	×
82	58	M	IV	1	no	—	×	×
83	53	F	IV	0	no	—	×	×
84	55	F	IV	1	no	—	×	×
85	48	F	IV	1	no	—	—	×
86	56	F	IV	0	no	—	—	×
87	74	M	IV	1	no	—	×	×
88	78	M	IV	0	no	—	×	×
89	68	F	IV	1	no	del(19)	—	×
90	69	F	IV	1	no	—	×	×
91	68	M	IV	0	no	—	×	×
92	64	F	IV	1	no	del(19)	—	×
93	56	F	IV	1	no	E709A/G719S(18)	×	×
94	49	F	IV	1	no	—	×	×
95	64	M	IV	1	no	—	×	×
96	77	M	IV	0	no	—	×	×
97	68	F	IV	0	no	—	×	—
98	64	F	IV	1	yes	—	×	×
99	48	M	IV	1	no	—	×	×
100	60	F	IV	1	no	—	—	—
101	66	M	IV	0	no	—	×	×
102	59	F	IV	1	no	—	×	×
103	72	F	IV	1	no	del(19)	×	×
