# Supplementary material for: EGFR Exon-Level Biomarkers of the Response to Bevacizumab/Erlotinib in Non-Small Cell Lung Cancer
Source: PLoS One. 2013 Sep 10;8(9):e72966. doi: 10.1371/journal.pone.0072966 (PMC3769372; doi:10.1371/journal.pone.0072966)
Supplement: Text S1 — Additional material and methods information. The first paragraph provides an extended description of the exon-level gene expression analysis. The second paragraph gives details about the assessment of the stability of the obtained results. (PDF) [file pone.0072966.s004.pdf]

EGFR exon-level biomarkers of the response to bevacizumab/erlotinib in non-small cell lung cancer

Florent Baty, Sacha Rothschild, Martin Früh, Daniel Betticher, Cornelia Dröge, Richard Cathomas, Daniel Rauch, Oliver Gautschi, Lukas Bubendorf, Susanne Crowe, Francesco Zappa, Miklos Pless, Martin Brutsche, on behalf of the Swiss Group for Clinical Cancer Research

Supporting Text S1

Material and methods

Exon-level gene expression analysis

Total RNA from bronchoscopic biopsy samples were extracted using miRNeasy Mini Kit (Qiagen) according to the manufacturer's recommendations and provided sufficient quality for microarray hybridization in 42 of 49 samples. Circulating RNA from peripheral blood samples was extracted using PAXgene Blood RNA Kit (Qiagen) followed by further purification on Qiagen RNeasy MinElute Cleanup Kit (Qiagen), and by the depletion of Alpha and Beta Globins using the GlobinClear Human Kit (Ambion) according to standard recommendations with the following exception: 5 μg RNA Cleanup kit (Zymo) was used for cleaning the depleted RNA instead of the magnetic beads provided in the Ambion kit, which significantly increased the recovery of RNA (80% versus 55% in our hands) and provided sufficient quality for microarray hybridization in all 75 samples. Total RNA were quantified using a NanoDrop ND-1000 and purity was assessed by determining the 260/280 ratio. Total RNA was monitored using the RNA Nano 6000 Chip on 2100 Bioanalyzer (Agilent) and RNA integrity number (RIN) was assessed for each sample. mRNA was hybridized on Affymetrix Human Exon 1.0 ST arrays (Affymetrix, Santa Clara, CA, USA) which contain approximately 1.4 M probesets. The hybridization was carried out as follows: target synthesis was led starting from 200ng total RNA (lung biopsies) or 350ng globin free total RNA (blood) using the WT expression kit (Ambion) following standard recommendations, except that 12 μg cRNA was used instead of 10 μg for entering the second reverse transcription. Fragmentation and labeling of amplified cDNA were performed using the WT Terminal Labeling Kit (Affymetrix). Synthesis reactions were carried out using a PCR machine (TProfessional, Biometra) in 0.2 mL tubes (Starlab, i1402-2008). 200 μL cocktail (25 ng/μL DNA) was loaded on Human Exon 1.0ST Array (Affymetrix) and hybridized for 17 hours (45°C, 60rpm) in Hybridization oven 645 (Affymetrix). The arrays were washed and stained on Fluidics Stations 450 (Affymetrix) by using the Hybridization Wash and Stain Kit (Affymetrix) under FS450_0001 protocol. The GeneChips were processed with an Affymetrix GeneChip Scanner 3000 7G. DAT images and CEL files of the microarrays were generated using Affymetrix GeneChip Command Control 3.0.0.1214. The exon and gene level probesets were pre-processed, quality checked and normalized using the RMA procedure.

Assessment of the stability of the obtained results

To assess the stability of our findings, a cross-validation strategy was used. Figure S2 left panel depicts the ROC curve based on the probeset showing the strongest association with TS12 (probeset 3002770). The best cut-off value, together with the corresponding false positive rate (FPR), true positive rate (TPR) and AUC are given. The re-sampling approach of .632 bootstrapping was used to assess the stability of our results. A series of new datasets was built from the original dataset, by sampling with replacement of the observations. The whole process, including feature selection and ROC analysis for the classification of BE responders was carried out on each newly created dataset. Figure S2, right panel shows the ROC curve obtained after bootstrap cross-validation (100 replications). The boxplots show the distribution of the TPR over the series of resampled datasets. The median area under ROC curve

obtained after bootstrapping procedure was 0.94 (95% confidence interval (CI): $0.70 - 1.00$). During the bootstrap procedure, the feature selection step revealed that in 71% of cases, either probeset 3002770 (48% of cases) or probeset 3002669 (23% of cases), showed the strongest association with TS12. Both probesets are located in the vicinity of the starting point of EGFR exon 18. In addition, we tested the aggregation of these 2 probesets around exon 18 by comparing their distance to the starting point of exon 18, with the distance of all other pairs of probesets available within EGFR (51 exonic probesets). The distance between the top 2 probesets and the starting point of exon 18 was significantly shorter than the distance between other pairs of probesets randomly chosen among the 51 available probes (permutation test: $p = 0.0016$).
